# Tracking the upstream history of aquatic microbes in a boreal lake yields new insights on microbial community assembly

**DOI:** 10.1093/pnasnexus/pgac171

**Published:** 2022-08-26

**Authors:** Sophie Crevecoeur, Yves T Prairie, Paul A del Giorgio

**Affiliations:** Département des Sciences Biologiques, Groupe de Recherche Interuniversitaire en Limnologie et en Environnement Aquatique (GRIL), Université du Québec à Montréal, Montréal, QC H2×1Y4, Canada; Département des Sciences Biologiques, Groupe de Recherche Interuniversitaire en Limnologie et en Environnement Aquatique (GRIL), Université du Québec à Montréal, Montréal, QC H2×1Y4, Canada; Département des Sciences Biologiques, Groupe de Recherche Interuniversitaire en Limnologie et en Environnement Aquatique (GRIL), Université du Québec à Montréal, Montréal, QC H2×1Y4, Canada

**Keywords:** aquatic microbial communities, microbial assembly, RNA:DNA ratio, rare biosphere, rank-abundance

## Abstract

Bacterial community structure can change rapidly across short spatial and temporal scales as environmental conditions vary, but the mechanisms underlying those changes are still poorly understood. Here, we assessed how a lake microbial community assembles by following its reorganization from the main tributary, which, when flowing into the lake, first traverses an extensive macrophyte-dominated vegetated habitat, before reaching the open water. Environmental conditions in the vegetated habitat changed drastically compared to both river and lake waters and represented a strong environmental gradient for the incoming bacteria. We used amplicon sequencing of the 16S rRNA gene and transcript to reconstruct the shifts in relative abundance of individual taxa and link this to their pattern in activity (here assessed with RNA:DNA ratios). Our results indicate that major shifts in relative abundance were restricted mostly to rare taxa (<0.1% of relative abundance), which seemed more responsive to environmental changes. Dominant taxa (>1% of relative abundance), on the other hand, traversed the gradient mostly unchanged with relatively low and stable RNA:DNA ratios. We also identified a high level of local recruitment and a seedbank of taxa capable of activating/inactivating, but these were almost exclusively associated with the rare biosphere. Our results suggest a scenario where the lake community results from a reshuffling of the rank abundance structure within the incoming rare biosphere, driven by selection and growth, and that numerical dominance is not a synonym of activity, growth rate, or environmental selection, but rather reflect mass effects structuring these freshwater bacterial communities.

Significance Statement Microbial communities are essential to the functioning of aquatic ecosystems. Common assumptions are that dominant microbes actively participate in microbial processes and that rare and dormant microbes can activate and become dominant under suitable conditions. However, the mechanisms dictating microbial community assembly are still under debate, and testing these assumptions requires a historical perspective. Therefore, we reconstructed the assembly of a lake microbial community by following the changes in relative abundance and activity of individual taxa from their source habitats upstream. Dominant taxa traveled mostly unchanged while rare taxa were the most dynamic and responsive to environmental gradient. This challenges the common view on community structure and draws attention to the importance of the rare biosphere in microbial assembly processes.

## Introduction

Microbial communities play a crucial role in aquatic ecosystems as they are the main transformers and recyclers of organic matter and nutrients (1). Their diversity of metabolism coupled with short generation time can lead to rapid changes in microbial community composition as environmental conditions vary (2). However, the mechanisms underlying those changes in community assembly are still poorly understood. In general, microbial community structures are characterized by a highly skewed rank-abundance distribution, with very few dominant taxa and a long tail of rare bacteria (3, 4). The study of this rare biosphere has been facilitated by the increased resolution of sequencing techniques (3). Based on 16S rRNA gene sequencing results, dominant taxa have generally been defined as the ones representing more than 1% of the community, whereas the rare biosphere are defined as taxa that fall below 0.1% of relative abundance (5). Numerically dominant taxa have generally been assumed to be favored by the local environmental conditions and therefore to be active and growing, likely responsible for the bulk of microbial processes (6). Rare taxa, on the other hand, have often been assumed to be less active or generally dormant, unresponsive (7), and less relevant for explaining ecological patterns (8, 9). Different reasons have been invoked to explain the maintenance of the rare biosphere: rare taxa adapted to only a specific kind of substrate or condition and with very limited growth potential, local taxa that are not growing and which compose a seedbank with the potential to activate when favorable conditions are met (5, 10–13), or new incoming taxa passively dispersed into the system and that remain mostly dormant (13, 14). However, there is increasing evidence that the rare biosphere may be more dynamic than once thought and play a disproportionate role in microbial processes (15–17) and on community structure and functions (18). There is clearly much still to be learnt regarding the mechanisms of community assembly that explain the distribution of rare and abundant taxa in natural microbial communities.

The mechanisms underlying microbial community assembly can only be efficiently captured when following community composition through space or time. For example, a study focusing on time series in various types of habitats have shown that rare taxa that become abundant [referred to as conditionally rare taxa (CRT), (19)] explained a substantial part of the community changes through time (19). Nevertheless, a follow-up study on soil bacteria reported that abundant taxa rather than CRT were the ones explaining community turnover (20). Additionally, studies of coastal sand and pelagic microbial communities indicated that very few members of the rare biosphere ever became dominant (21, 22) and had minimal contribution to biogeochemical cycles (21). In freshwater ecosystems, highly connected networks make the use of spatial history particularly relevant to study microbial community assembly (23). For example, studies across highly connected freshwater networks in the Arctic or boreal landscapes have reported that microbes in upslope environments such as soils (23–25), glacial rivers (26), or even snow banks (27) can become numerically dominant in downstream rivers and lakes through selection, may persist as dominant through passive transport (mass effects), or become part of the aquatic rare biosphere (28).

To the best of our knowledge, no study so far has linked the change in the relative abundance of individual taxa to their change in activity. This dimension is key to understanding microbial community assembly and distinguishing passive dispersal and mass effects from environmental selection and reactivity to the environment, which are difficult to disentangle based on abundance patterns alone (29). The 16S rRNA gene and transcript allow to follow changes in the relative abundance of individual taxa and to assess their reactivity to the environment by measuring changes in the RNA:DNA ratio (30). Although the RNA:DNA ratio can be subject to some biases [discussed in Steven et al. (2017) (31)], it is an overall robust indicator of microbial activity when compared to other metrics of cellular activity (32, 33). This approach has revealed unexpected levels of activity of taxa within the rare biosphere (34), sometimes exceeding the ones of dominant taxa (35, 36), challenging some longstanding assumptions. Another important piece of information often missing when assessing microbial community assembly with 16S rRNA gene is the link with functional traits. Yet, it has been shown that the number of ribosomal RNA operon (rrn), containing notably the 16S rRNA gene, is correlated with certain cellular processes such as growth rate and resource utilization (37, 38), so that bacteria carrying fewer rrn copies have in general a slow growth rate but efficient resource use, while higher rrn copies translate into a rapid growth rate but low growth yield (biomass produced by unit of substrate) (39). Therefore, by estimating 16S rRNA gene copy number, it may be possible to link the observed patterns of dominance and rarity with ecological strategies.

Understanding how a certain microbial community structure comes to be, not just in terms of its taxonomic composition but also how its taxa are distributed within a certain rank abundance shape, therefore requires a historical perspective that must include how taxa shifted in numerical abundance in time within a given habitat and also in space as communities disperse between habitats. History necessarily involves tracing both numerical changes and changes in activity, because there may be numerical shifts that are driven by mass effects, and others that are mediated by local recruitment, selection and therefore growth of taxa. This consideration of dispersal and activity is particularly important for our understanding of microbial communities inhabiting freshwater ecosystems, which typically form complex networks of highly interconnected habitats, where upstream history is a critical determinant of community assembly at any given point (23).

Here, we present a study where we reconstruct the processes that lead to the observed rank abundance structure of the microbial community of a typical oligo-mesotrophic boreal lake (Lake Simoncouche). This lake has a single main tributary that is responsible for over 90% of water inputs, which results in a relatively short water residence time and a system that is strongly influenced by external inputs. The riverine water flows through a dense macrophyte bed before discharging into the open lake waters. Environmental conditions vary greatly within this aquatic vegetated habitat relative to the river and the lake, representing both an environmental filter for the incoming riverine bacteria and a potential source of new bacteria to the lake. Reconstructing the assembly of the microbial community structure in this lake therefore implies assessing how these riverine communities shifts during their passage through the vegetated habitat and subsequently onto the lake, in terms of both numerical abundance and activity. In this study, we used high throughput sequencing of the 16S rRNA gene and transcript to follow the changes in relative abundance and activity of individual taxa, in order to understand the mechanisms underlying the assembly of the lake microbial community. In addition, this historical reconstruction allowed us to reassess some of the common interpretations that are made from static observations of rank abundance structures of microbial communities, such as the emergence and ecological implications of numerical dominance or the origins and persistence of the rare biosphere.

### Conceptual framework

**Fig. 1.**
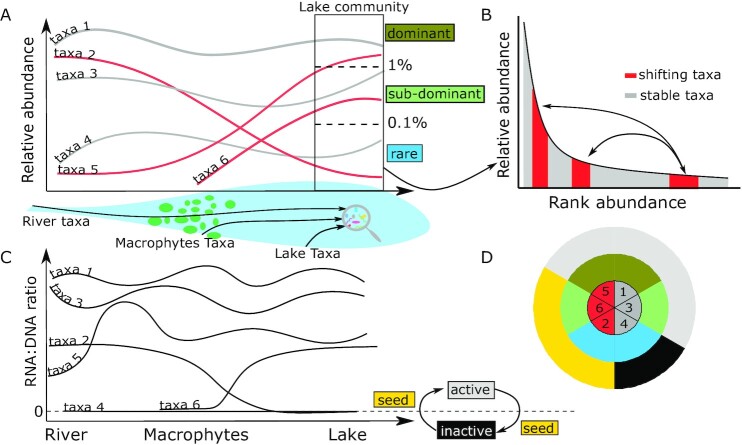
Conceptual scheme of the changes in relative abundance and activity of the lake microbial community. Individual taxa travel from the upstream river to the lake where the lake community is composed of dominant, subdominant, and rare taxa (A). Some of those taxa may have substantially shifted in relative abundance from the river and/or the vegetated habitat (macrophyte) to the lake and become dominant and vice versa (B). Those changes in relative abundance are also accompanied by changes in activity (C), and depending on their RNA:DNA ratio, taxa can be considered active (RNA:DNA ratio > 0), inactive (RNA:DNA ratio = 0), or seed that oscillate between null and nonnull ratios, which give rise to a complex lake community composed of taxa that each have their own history. (D) Here, the outer pie correspond to panel C, the middle pie to panel A, and the inner pie to panel B, where figures correspond to taxa numbering in panels A and C.

In order to understand the formation of the rank-abundance structure of the lake microbial community, we retraced the patterns in relative abundance and RNA contents of individual lake taxa from their source upstream to the lake (Fig. 1A). At any given time, the pelagic lake microbial community is composed of taxa that derive from a combination of sources: some are being transported by the inflowing river, additional taxa are recruited as the riverine water flows through the vegetated macrophyte-dominated habitat, and yet other taxa are recruited within the lake itself from various sources (surrounding soils, sediments, atmospheric deposition, and rainfall) (Fig. 1A). The distribution of these taxa into the abundance categories that are observed in the lake community reflects in part the successive rearrangements of the rank abundance structure of the source communities (river, vegetated habitat) plus the additional selection that occurs within the lake habitat. We operationally categorize the lake taxa according to their relative abundance in the lake: dominant with high relative abundance (>1%), subdominant (<1% and >0.1%), and rare with low relative abundance (<0.1%). Yet, the relative abundance of these taxa may have shifted drastically during transit from their riverine or vegetated habitat origin (Fig. 1B). For example, rare taxa in the river may become dominant in the lake and vice versa. On the other hand, taxa may stay relatively numerically stable all along the river-vegetated habitat-lake continuum. Because of the changes in environmental conditions in the vegetated habitat and then in the lake itself, we expect at least some components of the microbial community to shift both in relative abundance and in activity during passage, the latter would be reflected in changes in their relative RNA contents. In order to assess whether individual taxa are reactive to the environment or not, we further categorized taxa based on their individual patterns in RNA:DNA ratio along the water continuum (Fig. 1C). When DNA was detected but RNA could not be detected, the taxa were categorized generically as “inactive” (RNA:DNA ratio = 0), because we do not know if those taxa are dead, dormant with the capacity to activate, or have extremely low an undetectable activity. On the other hand, all taxa that were detected in both DNA and RNA were considered to have some level of cellular metabolism (RNA:DNA ratio > 0) and categorized as active. Finally, the taxa that shifted from undetectable (RNA:DNA = 0) to detectable in RNA (RNA:DNA > 0) were referred to as “seeds,” as they appeared to activate or inactivate depending on local environmental conditions. The lake community is therefore composed of taxa that each have their own history (Fig. 1D). With this framework, we were able to (1) identify the fraction of the lake community that can be retraced to upstream communities versus local recruitment, (2) reconstruct the patterns in numerical abundance of upstream lake taxa along the river/vegetated habitat/lake continuum, to infer underlying mechanisms (mass effects versus selection) of lake community assembly, (3) link these numerical patterns to patterns in RNA:DNA ratios to infer reactivity to environmental shifts and thus further discriminate selection versus passive transport, and (4) identify recruitment of taxa from local seedbank and differentiate this from random occurrence of maladapted taxa.

Our study site represents a detailed exploration of a small-scale version of environmental gradients and transition scenarios that are widespread across aquatic networks, and our goal is to derive insight on patterns and mechanisms of microbial community assembly that generally apply to these scenarios, rather than to extrapolate the actual numerical results we obtained to other systems.

## Results

### Physico-chemical parameters and bacterial community diversity and structure along the aquatic continuum

Environmental conditions changed, most of the time significantly, in the vegetated habitat compared to the river and the lake conditions (Fig. S1). Some variables, such as DOC, oxygen, pH, and temperature, tended to decrease in the vegetated habitat and then increase in the lake, while other variables such as phosphorus, chlorophyll a, and methane tended to increase within the vegetated habitat relative to both the incoming river and the downstream lake. Trends in nitrogen concentration differed as a function of the season but always showed a distinction between the vegetated habitat and the other habitats. Finally, conductivity seemed in general to continuously increase from the river to the lake. There was no global trend in alpha-diversity metrics between the different habitats, both Shannon and Simpson indices were significantly higher in the lake during late-July, but significantly lower in the lake in September. Operational taxonomic units (OTUs) richness was significantly lower in the lake during early and late-July (Fig. S2).

**Fig. 2.**
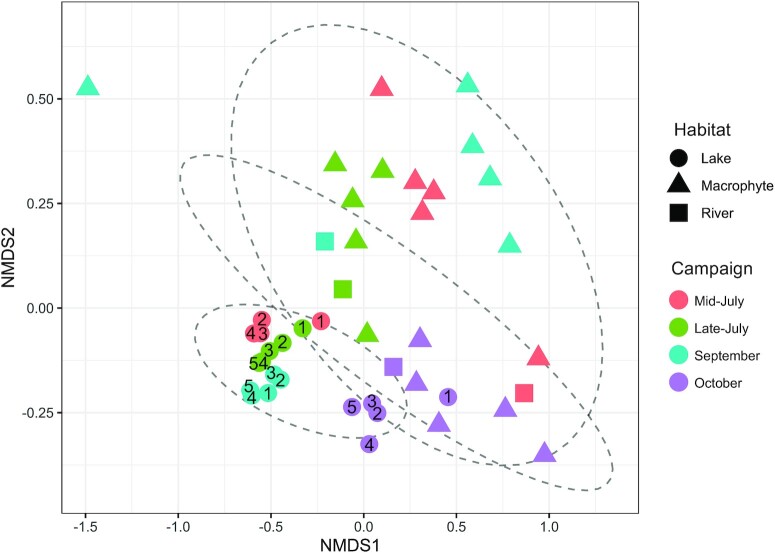
Nonmetric multidimensional scaling of the microbial community of Lake Simoncouche (based on the 16S rRNA gene) in the different habitats encircled by 80% CI ellipses, and colored as a function of the campaign. Lake samples are numbered from the point closest to the inlet (1) to the point closest to the outlet (5). Stress = 0.1.

Even though the water residence time within the vegetated habitat was relatively low (average of 1 to 5 d depending of the season), there was a clear spatial succession in the bacterial community structure along the river/vegetated habitat/lake continuum, where the lake bacterial community was clearly distinct from that of both the river and the vegetated habitat, and also much less variable than either of these (Fig. 2), despite the lake samples having been collected within a larger area (Fig. S3). There was also an apparent temporal clustering across the four campaigns for the river and vegetated habitats samples, as well as between October and the other campaigns for the lake, and samples within the latter habitat were clearly more tightly clustered. Although there was a modest directional succession in community structure from the inlet to the outlet within the lake, and evidence of some degree of seasonality, the lake samples tended to cluster closely together, suggesting that the lake community was shaped early on when entering the lake past the vegetated habitat and subsequently maintained, at each season. The lake community is clearly not the sum of the river and the vegetated habitat communities, but rather an emergent structure that results from both the reshuffling of the incoming communities and the addition of new components, as we describe below.

### Patterns of lake community assembly: categories of taxa linked to their source and activity

**Fig. 3.**
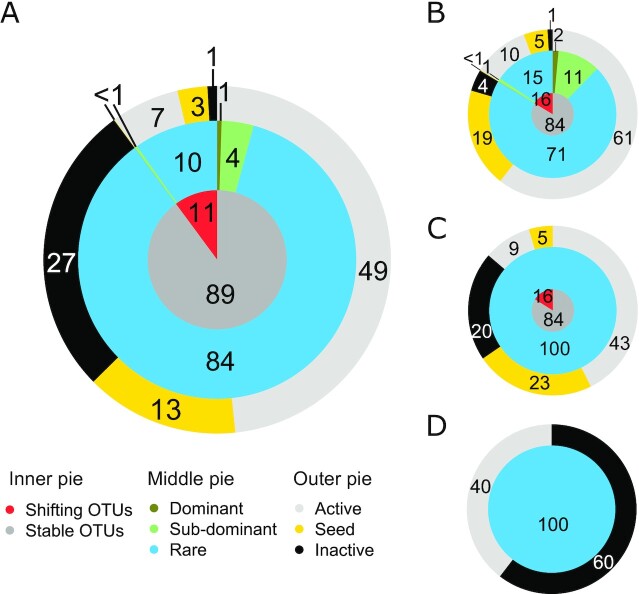
Average proportion of lake OTUs across the four campaigns shifting or staying stable in relative abundance (inner pie), overlaid with their categories of relative abundance in the lake (middle pie) and their changes in RNA:DNA ratio (outer pie) for the lake community (A), and details of lake OTUs first identify in the river (B), in the vegetated habitat (C), and in the lake (D). Proportions of the middle and outer pie are relative to the fractions in the inner pie. Numbers indicate the average percentages of lake OTUs. Only categories accounting for more than 0.1% of the lake OTUs are shown.

On average for the four campaigns, the lake microbial community was composed of 844 ± 200 OTUs originating from the river and its upstream network, 760 ± 529 OTUs first detected in the vegetated habitat, and 773 ± 302 OTUs only found in the lake. The description below focuses on the average percentage of lake OTUs across the four sampling campaigns. First, the vast majority of the lake OTUs (89% ± 3%) that could be retraced to the river or vegetated habitat communities remained stable in terms of abundance (according to the criteria presented in the “Methods” section) relative to their source community (Fig. 3A), and the majority of those OTUs was rare (84% ± 4%), yet there was a small number of dominant (1% ± 0.3%) or subdominant taxa (4% ± 2%) that remained stable as well. These dominant or subdominant stable taxa originated exclusively from the river (Fig. 3B), whereas all the new OTUs recruited within the vegetated habitat (Fig. 3C) and within the lake itself (Fig. 3D) remained within the rare category. There was therefore only a small proportion of OTUs shifting more than ten-fold in relative abundance (11% ± 3%) of river or vegetated habitat origin composing the lake bacterial community. Those shifts in relative abundance all occurred either within the rare category, or more seldom, shifted from rare to subdominant and vice versa. The only exception is one OTU in the September campaign that shifted from being subdominant in the river to dominant in the lake (not shown in Fig. 3 since this OTU accounted on average for less than 0.1% of the lake OTUs). Otherwise, during the four campaigns, no OTUs shifted from being rare in the river to being dominant in the lake, and only 4 OTUs (1 in the September and 3 in the October campaign) shifted from being dominant in the river to rare in the lake, which represented only 0.03% of the lake OTUs.

More than half of all the lake OTUs (regardless of source) were detected in RNA and considered active (56% ± 5%) and this comprises all the dominant and subdominants from riverine source, with the exception of 0.1% ± 0.1% of subdominant OTUs that was rare in the river and became subdominant in the lake, and behaved as seeds. The remainder of the rare OTUs that were detected in the lake could either be retraced to a seed behavior (16% ± 2%) or were not detectable in RNA and considered inactive (28% ± 2%), and the majority of the latter were OTUs that originated within the lake itself (Fig. 3D). OTUs shifting in relative abundance along the environmental continuum were for the most part active, although some also behaved as seeds, moving from being detectable to not detectable in RNA or vice versa.

Although the percentages linked to the different categories are averages for the four campaigns, these patterns were highly consistent across the individual campaigns (Fig. S4), as evidenced from the relatively low standard deviation (SD) for each category of taxa. The dominant taxa contributed on average to 57.5% of the community reads, whereas the subdominants and rare contributed 29.5% and 13%, respectively, and these proportions did not vary substantially between campaigns, suggesting that the rank-abundance structure of the lake bacterial community was relatively stable across summer and fall.

**Fig. 4.**
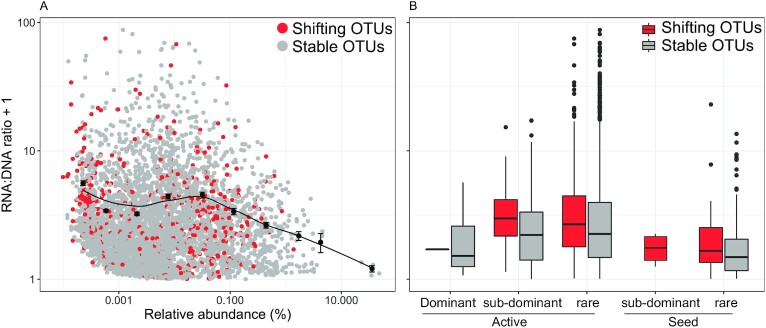
Relationship between the RNA:DNA ratio and the relative abundance of each OTUs in the lake across all campaigns with shifting OTUs colored in red and stable OTUs in grey where dots and error bar represent the means and the SE of the binned data, respectively. Smoothing line was drawn with the “loess” method (A). Average RNA:DNA ratio in the lake for each categories of taxa where the middle line inside each box plot is the median, the box delimits the 25th and 75th percentiles, the whiskers extend to the 1.5 *inter-quartile range, and the individual dots are outliers (B).

There was an overall weak negative relationship between the RNA:DNA ratio and the relative abundance of taxa, with increasing scattering in ratios towards the rarer taxa (Fig. 4A). Among the active but stable fraction, the ratio increased on average from the dominant to rare OTUs, and the OTUs shifting in relative abundance had on average a higher RNA:DNA ratio for each of these categories (Fig. 4B). The dispersion of the ratio around the mean also increased from dominant to rare OTUs. The seed taxa had on average lower RNA:DNA ratios than the active taxa, whether they were stable or shifted in numerical abundance (Fig. 4B).

### Categories of taxa linked to average gene copy number

For all the OTUs for which 16S rRNA gene copy number could be estimated (68% ± 2% of the OTUs on average), there was a significant difference in the 16S rRNA gene copy number between the categories of relative abundance (dominant, subdominant, and rare) (Kruskal–Wallis *P* < 0.01) with dominant OTUs having on average 1.8 copy of 16S rRNA gene and subdominant and rare OTUs 2.4 and 2.6 copies of 16S rRNA gene, respectively. Pairwise tests confirmed significant differences between dominant and subdominant OTUs (Pairwise Wilcoxon Rank Sum Tests *P* = 0.01), and rare OTUs (Pairwise Wilcoxon Rank Sum Tests *P* < 0.01) but not between subdominant and rare OTUs. There was no difference of 16S rRNA copy number by campaign, but when tested separately, only the September campaign showed a significant difference between 16S rRNA gene copy number as a function of OTUs relative abundance (Kruskal–Wallis *P* < 0.01), and the difference was also only between dominant and subdominant OTUs (Pairwise Wilcoxon Rank Sum Tests *P* = 0.05) and rare OTUs (Pairwise Wilcoxon Rank Sum Tests *P* = 0.02). There were no significant differences in 16S rRNA copy number between shifting and stable OTUs.

## Discussion

Even though the water only resides from 1 to 5 days within the vegetated habitat, the lake bacterial community was clearly distinct and less variable from that of the river and the vegetated habitats, where both habitats acted as the primary sources of lake taxa. In this regard, the lake acts as a funnel, selectively retaining and excluding incoming taxa, and in the case of the rare bacteria, often shifting their relative abundances. Within each habitat, there was also a temporal succession that was more variable for the river and the vegetated habitat than for the lake, where it was directional and much more clearly defined, probably because of the overall higher number of rare OTUs in the river and vegetated habitat compared to the lake. This pattern can be explained by the stronger influence of terrestrially derived taxa generally in river sites (25) and the highly heterogeneous nature of the vegetated habitat studied here, with patches of two different species of macrophytes. On the other hand, the lake pelagic habitat is characterized by more homogenous conditions, and longer residence time, which favors deterministic taxa selection (40). It is interesting to note that the lake community is determined almost immediately upon the passage to open lake waters from the vegetated habitat and subsequently maintained. Within the lake itself, only relatively small changes were observed, suggesting that the environmental filters and other processes that shape the lake community are operating at very small spatial and temporal scales.

Numerous studies have followed microbial community composition through space and time in order to assess changes in community structure, identify the main environmental drivers of these changes and unveil the dynamics underlying microbial community assembly (19, 21, 22, 24–27, 41, 42). Nevertheless, the observed changes in relative abundance of taxa have seldom been linked to changes in their degree of activity, which is often the missing piece of information to understand the underlying mechanisms of community assembly (23, 29). Here, we have reconstructed the source of taxa and the processes that lead to their observed rank abundance and activity distribution in a typical boreal lake bacterial community, as a concrete case example of aquatic bacterial community assembly. Lake Simoncouche receives most of its water and material inputs from a single inflow, this reconstruction involves following the changes in relative abundance and activity of riverine microbes through a well-defined aquatic continuum characterized by sharp environmental gradients as they enter the lake, which allows to define the upstream history of the lake bacterial communities. In this regard, our study site represents a small-scale version of environmental gradients and transition scenarios that are widespread across aquatic networks, and the insight on patterns and mechanisms of microbial community assembly that we derive here should generally apply to these scenarios, even if the numerical results themselves cannot be extrapolated to other systems.

We used the number of reads of RNA normalized to DNA reads per taxon (RNA:DNA ratio) as an indication of microbial responsiveness to the environment since, in active cells, the number of ribosomes is expected to change as a function of cell growth and metabolic rates, which are themselves driven by local environmental factors much more than the genome copy number (43, 44). Although ribosome accumulation has been observed in some dormant bacteria (45, 46), dormancy and low activity are generally associated with low ribosomal contents (30). Some Bacteria, SAR11 for example, can be metabolically active despite their consistently low number of ribosomes (47). The use of the RNA:DNA ratio as an actual proxy of the level of activity is somewhat problematic (31), because for any given growth rate or metabolic rate, the RNA:DNA ratio levels may actually differ between taxa (48). Historically, a threshold of 1 for the RNA:DNA ratio has been proposed to distinguish between active and dormant cells (30), but this has led to an overestimation of the number of dormant bacteria (31). In this study, we used a very conservative threshold of 0 to differentiate active from inactive taxa, such that taxa with undetectable levels of RNA were assumed to be inactive. We acknowledge that this is a purely operational discrimination, and that bacteria with ratios = 0 may be active and vice versa, but our approach is not based on the absolute values of the ratio, but rather on the consistency in the patterns of the RNA:DNA ratio of individual taxa along the aquatic continuum. For example, we discriminated seed taxa as those whose ratio changed from zero to more than zero or vice versa during transit and that therefore expressed the ability to activate or inactivate depending on environmental conditions; we classified taxa as “inactive” as those whose ratio was consistently = 0, and our assumption is that these taxa have consistently low activity or are dormant. Likewise, we classified taxa as “active” as those having a ratio consistently >0 along the entire continuum, and made no assumption regarding their level of activity, although we do find systematic differences in the average ratio between the different abundance categories that we identify.

Our results represented the average or compiled data from four sampling campaigns that took place from mid-July to late October. Although we expected patterns observed during the summer to differ from the fall, we noticed a very high consistency in the proportion of each category of OTUs across the four campaigns (Fig. S4). The fact that those patterns of assembly seemed to always be the same regardless of the season suggests that there is a certain consistency in the rules of assembly that transcends seasons and hydrological scenarios.

### Dominant taxa are generalists with broad tolerances

We observed that all dominant lake OTUs were already dominant in the inflowing river community, and no rare OTUs from the river or within the lake was observed to shift in relative abundance to become dominant in the lake. The dominant OTUs in the lake microbial community represented a very small proportion (1%) of the total number of OTUs detected in the lake, which is consistent with the structure of the rank-abundance curve observed in many aquatic systems (10, 29). Previous studies of marine microbial communities over time also observed that the dominant phylotypes were generally stable in time (49, 50). This suggests that the dynamic aspect of community reassembly might not necessarily involve rare taxa becoming dominant and vice versa.

Dominant OTUs in our system had on average a lower and more stable RNA:DNA ratio than subdominant and rare OTUs, suggesting a relatively low level of activity and degree of responsiveness to changes in environmental conditions. The fact that the dominant OTUs were present in relatively high abundance all along the river/vegetated habitat/lake flow path without expressing either large numerical changes or major shifts in terms of RNA in response to the drastic changes in environmental conditions suggests that these OTUs may be rather generalists with a wide range of resource preferences and environmental tolerances. In addition, some of these OTUs appeared to be also broadly distributed, because they belonged to typical freshwater genera like *Limnohabitans, Polynucleobacter, Planktophila*, and *Synechococcus* (51). When it comes to the processes of community assembly, the influence of local and regional factors on bacterial communities still needs to be disentangled (52). In our case, the passage of these OTUs through the vegetated habitat and onto the lake, without major changes in relative abundance, would suggest the predominance of mass effects, when microbial dispersal rate is higher than the rate at which the local environment selects, resulting in a passive transport of cells from their source environment (40). This process prevails in systems with short residence time (26, 41). Moreover, an assessment of the changes in relative abundance within the lake indicated that only 3% ± 1% of the lake OTUs shifted by more than ten-fold in relative abundance when travelling down the lake. However, mass effects alone are unlikely to explain the persistence of the dominance of these taxa within the lake, since the lake water residence time (average of 50 d) would provide ample opportunity for other taxa to grow and numerically take over. In order to maintain numerical dominance in the lake community, these taxa must maintain some level of growth, yet the consistently low number of 16S rRNA gene copy for the dominant OTUs suggests that these taxa may have an intrinsically slow growth rate, but may be more efficient when facing either shifting or limiting resources than other taxa (37). Therefore, even though those dominant OTUs appeared to be relatively unresponsive to environmental shifts, with a numerical distribution that at times may be explained by dispersion and passive transport, their ecological strategy of broad tolerances (leading to high capacity to persist under very different environmental conditions), slow growth rates but efficient resource utilization under shifting conditions, seems to be the key features that leads to dominance in the aquatic systems studied here. This perspective on dominant OTUs represents somewhat of a departure from the traditional view of microbial dominance as generally associated with fast growth, high turnover, and a high degree of responsiveness to environmental changes (6).

### Rare taxa may be highly responsive to the environment

The assembly processes of the rare biosphere are still unclear and there is currently a lack of proper characterization of the rare biosphere and of the different types of rarity. Snapshots of microbial community composition identify the rare biosphere as the long tail observed in the rank-abundance curve (3), but rarity can also be habitat or time specific (29). Based on fluctuations of taxa abundances in times, Lynch and Neufeld (2015) (12) proposed a scheme in which taxa can be classified as permanently, transiently (immigrating to the community), or conditionally rare (becoming dominant if conditions change), the latter already defined by Shade et al. (2014) (19). We believe those temporal categories of rarity can be transposed to a spatial context. For example, in our case, the majority of the rare OTUs found in the lake had transited the aquatic continuum or had been present in the lake without major shifts in relative abundance and could therefore be categorized as “permanently rare.” There was also a high number of transiently rare taxa in the river (1533 ± 831) and vegetated habitat (1617 ± 454) that never reached the lake and therefore were not taken into account in our analysis. Those transiently rare taxa seemed to follow a stochastic principle of assembly as they are brought by immigration and can be lost by dispersion (29). Regardless, it is clear that this transient category accounted for a large number of rare OTUs whose detection may be related to a combination of stochastic transport and detection thresholds. Finally, only one OTU across the four campaigns had a behavior that was similar to a “conditionally rare taxa” that became dominant in the lake, but was not even recruited from the rare biosphere, as it was rather subdominant in the river. We acknowledge that our spatial framework might be too limited to observe conditionally rare taxa, although the shifting environmental conditions within the vegetated habitat would have expectedly triggered a response in the microbial community, but in our case, this response occurred mostly within the rare biosphere, as we discuss in sections below.

Adding the dimension of activity to the environment, as reflected in the patterns of RNA:DNA ratio, called for additional categories of rarity to be considered. Within the broad category of permanently rare, there were at least three distinct groups of rare taxa: those that were never detected in RNA, which we assumed to be either dormant or with extremely low levels of activity; those that were consistently detected in RNA, which we assumed to be active, but which had highly varying levels of activity; and those that shifted from being detectable to nondetectable (or vice versa) in RNA, which we referred to as seeds, and which we discuss in a separate section below. The permanently rare but consistently “inactive” taxa differed from the “transiently rare” taxa in that they were consistently detected, yet showed no apparent reactivity to the environment. We have generically referred to OTUs that were undetected in RNA as being “inactive,” but we do not know if these cells are dead, dormant, or even active but extremely slow growing. For the purpose of this study, however, what is relevant is the change of state, i.e. from undetectable to detectable, of a given OTU along the continuum, which we assume to reflect reactivity to the environment. It is interesting to note that most of the permanently rare and consistently inactive taxa were actually first detected within the lake itself (Fig. 3D), and fewer originated from the vegetated habitat (Fig. 3C), and even fewer from the river (Fig. 3B). This would suggest that these may be maladapted taxa (53) with a limited capacity to persist (and thus the declining % originating further upstream) but that are nevertheless regularly transported into the lake pelagic zone likely from proximal sources, such as immediately adjacent soils or even from sources within the lake, such as fish or other distinct habitats.

The permanently rare OTUs that were consistently detected in RNA comprised a remarkably large fraction of the overall rare assemblage of the lake, and although these taxa remained at low abundance, they were extremely dynamic in terms of their RNA contents along the aquatic continuum. The significant higher number of 16S rRNA gene copy for subdominant and rare taxa suggests that OTUs in those categories may have a relatively high growth rate coupled with narrow resource preferences (37), which are also a characteristic of specialist taxa (54). As a matter of fact, specialist taxa are known to locally peak in abundance, respond more to environmental conditions, and be subject to habitat filtering at a macro and micro-scale (54). Although we did not observe local peaks in relative abundance, the increase in RNA:DNA ratio and ratio variability as relative abundance decreases corroborates the idea that these taxa have higher sensitivity to changes in environmental conditions and habitat filtering. There is additional evidence of disproportional activity of the rare biosphere (15, 30, 35, 36, 55) and even transcriptionally active rare taxa that display no growth (56). Even if they did not peak in relative abundance, rare taxa seemed therefore to potentially play an important role in microbial processes given their high activity. Their rarity seemed, for the most part, due to strong external pressure (for example competition, grazing, or viral lysis) rather than a maladaptation to their local environment. Therefore, this category of rare taxa may be assembled in a more deterministic manner, driven by environmental and biological factors that may favor their activity but also suppress their abundance (29). These latter may include selective grazing (57), for example demonstrated for the aerobic anoxygenic phototrophic (AAP) bacteria that, in spite having higher growth rate than the bulk bacterial community, have consistently low abundances due to top down control (58); viral infection that can be selectively directed toward certain taxa or highly active cells (59); or strong competition for resources (60).

### A high proportion of taxa are recruited locally and form the seed bank

The concept of seed bank is inextricably linked to dormancy, since a seed bank is defined as a reservoir of dormant cells that have the potential to activate and grow when environmental conditions become favorable (10, 61). Here, we considered seeds, those OTUs that shifted from undetectable (RNA:DNA = 0) to detectable in RNA (RNA:DNA > 0) as they transited through the river-vegetated habitat-lake continuum. We did not, however, define OTUs with a null ratio as dormant, unlike in Jones and Lennon (2010) (30), and rather used a more stringent definition by just considering them inactive. We found that seeds comprised over 15% of the lake microbial community. Interestingly, all the seed taxa that activated within the lake remained rare, suggesting that the environment might still be suboptimal, or that they cannot compete with the dominant taxa already established due priority effect (25, 62).

Regardless of their behavior in terms of RNA, many rare OTUs found in the lake were apparently recruited from the vegetated habitat or from within the lake itself, and were not detected in the river, and in this regard, these taxa belonged to the broader definition of seed bank as they greatly increased the extant pool of genetic diversity (10). In the ocean, studies have found that the seed bank was not composed of migrant taxa but rather of taxa that were always present but often in extremely low abundance (63, 64). In our case, it is also possible that the OTUs originating from the vegetated habitat and from within the lake were also present in the river but in extremely low and undetectable abundance. To explore this possibility, we retraced these new distinct lake OTUs in the deep sequencing results of samples taken during the late-July campaign. We found that only 7% of the distinct lake OTUs could be retraced back to the deep sequencing sample of the vegetated habitat, and to only 1% of the river deep sequenced sample (Fig. S5). In addition, 12% of the new OTUs originating in the vegetated habitat could be retraced to the deep sequencing sample of the river (Fig. S6). Therefore, it seems rather unlikely that the new taxa detected in the lake were always present in the river or in the vegetated habitat but in extremely low abundance. There appears to be systematic differences in the rare assemblages of these habitats that are not simply a consequence of sequencing depth but must reflect local recruitment and selection.

### Shifts in relative abundance mostly happen within the rare biosphere

Taxa that shifted substantially in relative abundance only represented a small proportion of the lake microbial community, and those shifts essentially occurred within the rare biosphere. Taxa that shifted in relative abundance within the rare assemblage correspond to what Lynch and Neufeld (2015) (12) call permanently rare with a periodic distribution. We do not know to what extent these shifts in the relative abundance of rare taxa are driven by growth or dispersal and mass effects, but we have shown that on average, the rare taxa that shifted tended to have higher RNA:DNA ratios than the nonshifting taxa. This suggests that the patterns in abundance may be driven by growth, and consistent with the observation that bacterial growth rate may be negatively correlated with density (65). Although RNA is not in itself a reliable indicator of cell growth (48), it still makes sense that OTUs substantially shifting in relative abundance would be associated to higher ribosomal contents, either to growth in the case of an increase in their relative abundance, or as a response to stress or other pressures in case of a drastic decrease in their relative abundance.

We acknowledge that our genomic analysis is based on relative abundance and does not allow us to capture change in absolute abundance for describing population dynamics (66). However, we used flow cytometry total cell counts collected for the September and October campaigns to address this bias. First, we observed no significant differences in flow cytometry cell counts across each habitat (anova *P* = 0.5 and 0.8, respectively) for each campaign. We then estimated the actual abundance of each taxon by multiplying the total cell count by its % read contribution (assuming that % of reads is analogous to % of cell abundance), and we recalculated the proportion of OTUs switching by more than ten-fold in absolute abundance. This yielded very similar results to the ones obtained with the relative read abundance, and the proportion of OTUs shifting in absolute abundance during the September and October campaign were the same than the one obtained with relative abundance (23% and 12%, respectively). These results suggest that the changes observed here in relative abundance seem to be consistent with what would be inferred from absolute abundances. As for changes in activity, we only discriminated between ratio > or = to 0, therefore multiplying transcript reads count by a quantitative measure (for example a qPCR assay) would not change the outcome of our analysis.

Overall, these observations challenge the view that the dynamics of the rank abundance structure mainly involves rare taxa becoming dominant and vice versa. Although the environmental gradients that shape the microbial communities that inhabit Lake Simoncouche are narrower compared to other possible aquatic gradients and ecotones, we believe that this case study does reflect many environmental scenarios commonly encountered along aquatic networks. In these scenarios, the bulk of the reshuffling of the rank abundance structure of aquatic microbial communities occurs within the rare assemblage itself, driven by a combination of mass effects, selection, growth, and loss, and numerical dominance is not maintained through high growth or reactivity to the environment.

## Materials and methods

### Study site and physicochemical parameters

Lake Simoncouche is located in the boreal region of Quebec, Canada (48° 13′ 52″ N, 71° 15′ 3″ W). The main tributary of the lake is a river flowing into an area densely colonized by emergent (*Typha sp*.) and floating-leaved (*Brassenia sp*.) macrophytes from spring to late fall (67). The rest of the lake is mostly open water with submerged macrophytes colonizing a few other shallow areas of the lake, with a water residence time of 50 d on average (68) [see Desrosiers et al. (2022) (67) and Vachon and del Giorgio (2014) (68) for more background information on the lake]. Four field campaigns were carried out during mid- and late-July (10–15, 24–29), September (18–23), and October (23–28) of 2016. During each campaign, study sites were chosen following the water continuum, i.e. one sample in the upstream river, five sites at different points within the vegetated habitat and five sites along a transect within the lake (Fig. S3), with the exception of the mid-July campaign for which only four lake samples were collected. Residence time within the vegetated habitat has been calculated by dividing the estimated volume of this habitat by the average inflow discharge during each campaign (see the “Supplementary Material” section). Temperature, conductivity, dissolved oxygen (DO), and pH were taken at each sampling site using a YSI multiparameter probe (Yellow Springs Instruments, OH, USA). Samples for physicochemical and molecular analysis were taken at the surface with a 2-L brown bottle and kept in a cooler until processed in the laboratory on the same day.

Water samples for DOC measurement were filtered through a 0.45 µm filter and analyzed with an OI1010 TOC analyzer. Samples for Chlorophyll a were filtered in glass fiber GF-C filtered and extracted with ethanol and concentrations were measured with a spectrophotometer. Samples for total phosphorus (TP) and total nitrogen (TN) measurements were digested with persulfate and alkaline persulfate, respectively, and then analyzed as described in Rasilo et al. (2015) (69). Methane partial pressure (pCH4 in µatm) was measured with the headspace technique as detailed in Rasilo et al. (2015) (69). For the third and the October campaign, bacterial abundance was measured from water samples preserved with 1% glutaraldehyde and analyzed with a FACSCalibur flow cytometer following del Giorgio et al. (1996) (70).

### Molecular analysis

Water samples were prefiltered through a 20-µm zooplankton mesh and then 250 ml of each sample were filtered through a 0.2 µm polycarbonate filter to collect microbial cells. The filter for DNA was stored at −80°C until extraction, and the filter for RNA was stored in RNA Later (Life Technologies) in the fridge overnight and then at −80°C until extraction. Filters were extracted with the Power Water DNA and RNA isolation kits (MoBio). A PCR for amplifying the 16S rRNA gene using the primers 515F (5′‐GTGCCAGCMGCCGCGGTAA‐3′) and 806R (5-′GGACTACHVGGGTWTCTAAT‐3′) was routinely run on the extracted RNA to verify it was free of DNA contamination, and then converted to cDNA with the High Capacity cDNA Reverse Transcription Kit (Applied Biosystems-Ambion). The 16S rRNA gene from DNA and cDNA samples was amplified with the same 515F and 806R primers and sequenced on an Illumina MiSeq2000 following a pair‐end approach at Genome Quebec Innovation Centre, Montreal, Canada. Additionally, one river, lake and macrophyte sample of the late-July campaign were sequenced again separately on an extra Illumina plate to produce deep sequenced samples for each of these habitats.

### Bioinformatic analysis

The 16S sequences were analyzed with the Uparse pipeline (71). Singleton and chimeras were removed and sequences were clustered into OTUs at ≥ 97% similarity. Taxonomic assignment of these OTUs was performed using the Mothur classifier (72) with a 0.8 confidence threshold based on the SILVA reference database (73). We obtained on average 77,643 (from 10,114 to 109,706) sequences, which comprises on average 88,323 (from 75,953 to 97,761) sequences in the river, 73,511 (from 10,114 to 109,706) in the vegetated habitat, and 77,592 (from 52,918 to 98,630) in the lake. The OTU table was rarefied at 10,114 reads per samples using the Qiime command multiple_rarefaction_even_depth.py (74). This process was repeated 100 times and the mean number of reads from the 100 rarefactions was used. Downstream statistical analyses were performed in R (75).

### Statistical analysis

Significant differences of environmental variables, alpha-diversity metrics (Shannon and Simpson indices, and OTUs richness; calculated with the R vegan package (76)) and flow cytometry counts across the different habitats were tested with a one-way anova, if anova assumptions were met, or else with a nonparametric Kruskal–Wallis test.

For each OTUs, the RNA:DNA ratio was calculated by dividing the number of cDNA reads by the number of DNA reads as in Charvet et al. (2014) (77). OTUs that were systematically not detected in DNA and for which no spatial pattern could be inferred were excluded from the analysis, even if those OTUs were detected in RNA [referred to as Phantom taxa in Bowsher et al. (2019) (33)]. For each campaign and for the habitats that had more than one sampling site (vegetated habitat and lake), the average relative abundance and ratio was calculated for each OTUs, in order to obtain one value per habitat. In some cases, OTUs were occasionally not detected in DNA or RNA despite being present elsewhere in the water continuum. In those cases, and in order to calculate the averages, the ratios of those OTUs were either set to 100 (detected in RNA but not in DNA) or to 0 (not detected both in DNA and RNA) as suggested in Bowsher et al. (2019) (33).

Lake OTUs were categorized as dominant (>1% in relative abundance), subdominant (< 1% and >0.1%), or rare (<0.1%). Lake OTUs whose relative abundance in the lake differed by more than ten-fold compared to the relative abundance in their source community (river or vegetated habitat) were considered to have substantially shifted in relative abundance (referred to as “shifting” OTUs), whereas the rest of the OTUs that did not change in relative abundance were referred to as “stable.” For assessing patterns of activity, OTUs with a RNA:DNA ratio above 0 in all three environments were considered active, whereas OTUs that had a ratio of 0 (no RNA detectable) all along were considered inactive. OTUs that shifted from undetectable (RNA:DNA = 0) to detectable in RNA (RNA:DNA > 0), and vice versa, were termed “seeds” as they appeared to have the potential to activate or inactivate. Finally, the average percentage of all the above-mentioned categories (shifting or stable in relative abundance, dominant, subdominant, or rare; and active, inactive, or seeds) was calculated across the four sampling campaigns and represented with a multilayer pie chart with the R package plotrix (78).

The scatter plot of the RNA:DNA ratio as a function of the relative abundance of each OTUs across all campaigns and the boxplot of the RNA:DNA ratio for each category of OTUs were represented with ggplot2 (79). The trend line in the scatter plot followed the average and SD of 13 bins of data grouped with the cut_number R command from ggplot2 (79). For those two plots, ratios set to 100 or to 0 due to undetected DNA or RNA were not accounted in the calculation as those values do not represent a true value but rather the absence of a value, and would cause an artificial trend in the scatter plot and the boxplot.

The average 16S rDNA copy number of all the OTUs was evaluated with the tool “estimate” of the Ribosomal RNA Database (80). Differences in 16S rRNA gene copy number were assessed with a Kruskal–Wallis test since the data did not follow a normal distribution, and variance was not homogenous amongst groups. Pairwise Wilcoxon Rank Sum tests with Bonferonni adjusting method was used to assess differences between groups.

## Supplementary Material

pgac171_Supplemental_File

## Data Availability

Sequences have been deposited on the GenBank database (accession PRJNA828579).
