# Supplementary material for: Tracking the upstream history of aquatic microbes in a boreal lake yields new insights on microbial community assembly
Source: PNAS Nexus. 2022 Aug 26;1(4):pgac171. doi: 10.1093/pnasnexus/pgac171 (PMC9802056; doi:10.1093/pnasnexus/pgac171)
Supplement: pgac171_Supplemental_File [file pgac171_supplemental_file.docx]

**Supplementary Information for**

Tracking the upstream history of aquatic microbes in a boreal lake yields new insights on microbial community assembly

Sophie Crevecoeur^a,b^, Yves T. Prairie^a^, Paul A. del Giorgio^a^

Sophie Crevecoeur

Email: [sophie.crevecoeur@ec.](mailto:sophie.crevecoeur@ec.)gc.ca

**This PDF file includes:**

Supplementary method

Figures S1 to S6 (not allowed for Brief Reports)

Supplementary Information Text

**Methods.**

Estimation of the vegetated habitat volume : Based on Desrosier et al., 2022 (68), the area of the vegetated habitat dominated by Typha latifolia, which correspond to the area sampled as the dense vegetated habitat in our study and in our Fig. S2, represents 6% of lake Simoncouche (which has a total area of 0.83 km2) and has an average depth of 1m.

vegetated habitat volume=6% X Lake area X average depth= = (6 X 830000 m^2 X 1m)/100=49 800 m^3

Residence time of the vegetated habitat is estimated by dividing the volume of the vegetated by the average lake discharge for each campaign (mid-July : 0.4 m3s-1, late-July : 0.5 m3s-1, September : 0.1 m3s-1, October : 0.4 m3s-1).

Vegetated habitat residence time = (vegetated habitat volume)/(average lake discharge)

Mid-July : =(49 800 m^3)/(0.4 m^3 s^(-1) )=124 500 s ≅ 1 day and 11h

Late-July : =(49 800 m^3)/(0.5 m^3 s^(-1) )=99 600 s ≅ 1 day and 4h

September : =(49 800 m^3)/(0.1 m^3 s^(-1) )=498 000 s ≅ 5 days and 18h

October : =(49 800 m^3)/(0.4 m^3 s^(-1) )=124 500 s ≅ 1 day and 11h


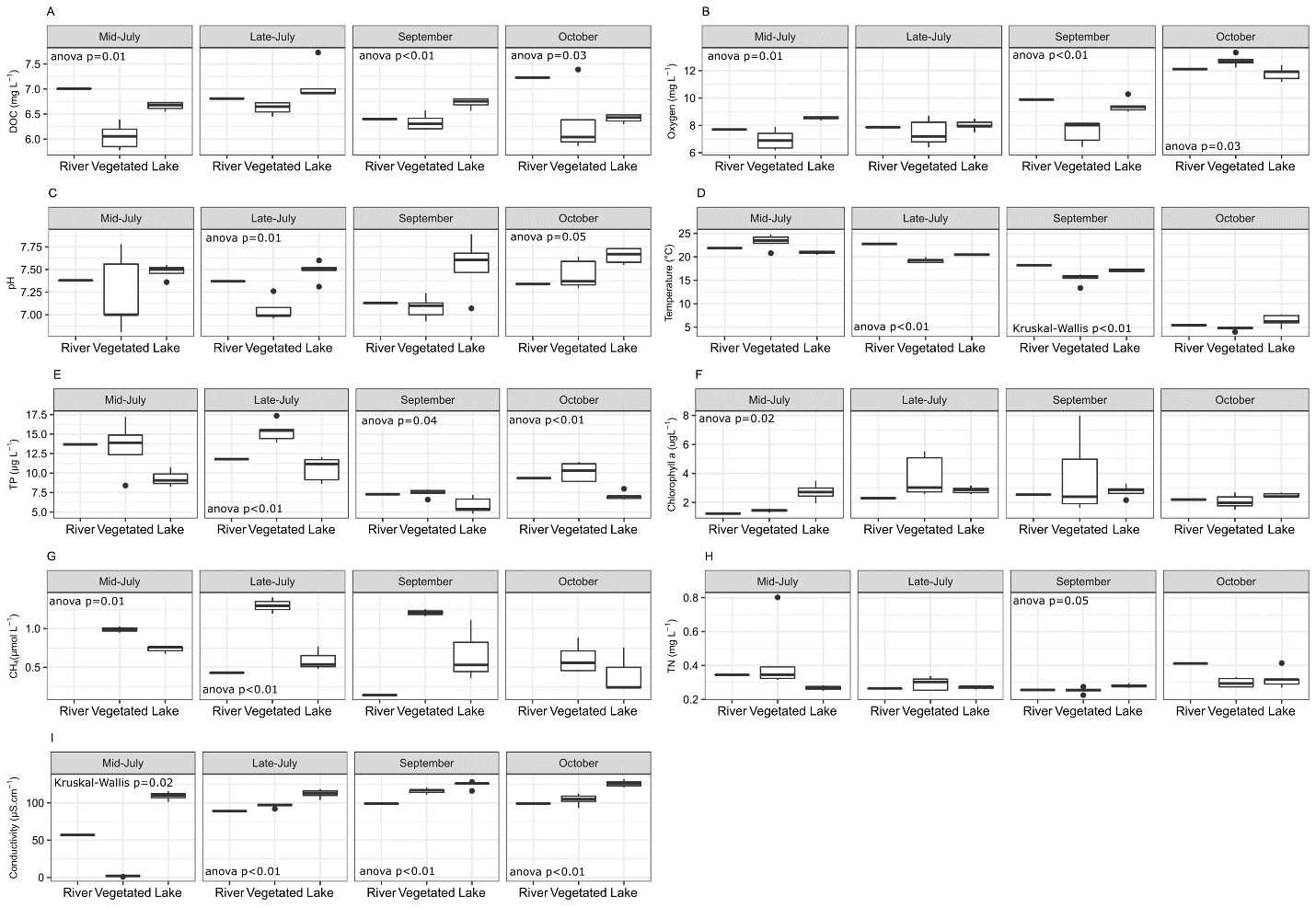


Fig. S1. Average values of DOC (A), oxygen concentration (B), pH (C), temperature (D), total phosphorus (E), Chlorophyll a (F), methane (G), total nitrogen (H) and Conductivity (I) in the different habitat. The middle line inside each box plot is the median, the box delimits the 25th and 75th percentile, the whiskers extend to the 1.5 * inter-quartile range, and the individual dots are outliers. When significant, differences of environmental variables across habitats are indicated by the name of the test and associated p-value.


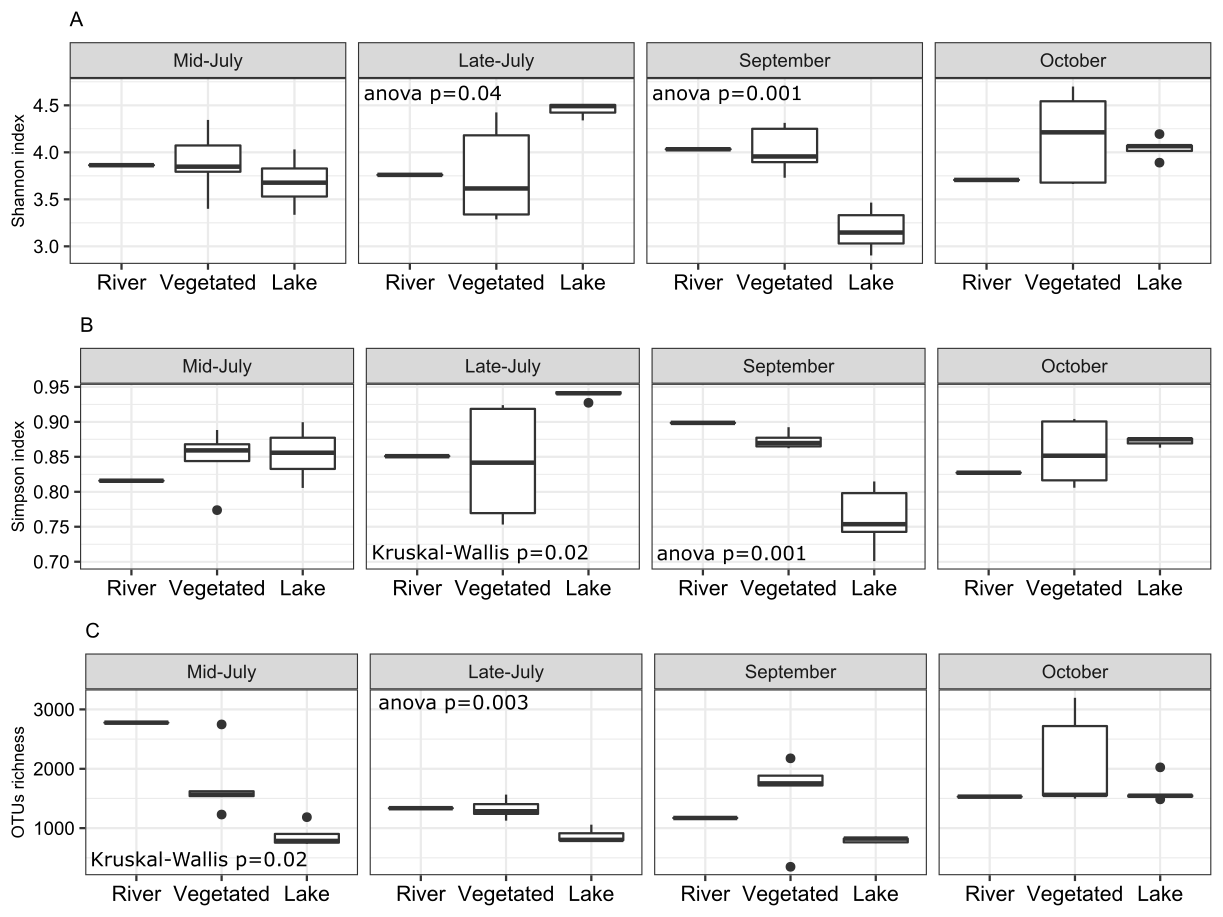


Fig. S2. Average values of Shannon index (A), Simpson index (B), and OTUs richness (C), in the different habitat. The middle line inside each box plot is the median, the box delimits the 25th and 75th percentile, the whiskers extend to the 1.5 * inter-quartile range, and the individual dots are outliers. When significant, differences of diversity indices across habitats are indicated by the name of the test and associated p-value.


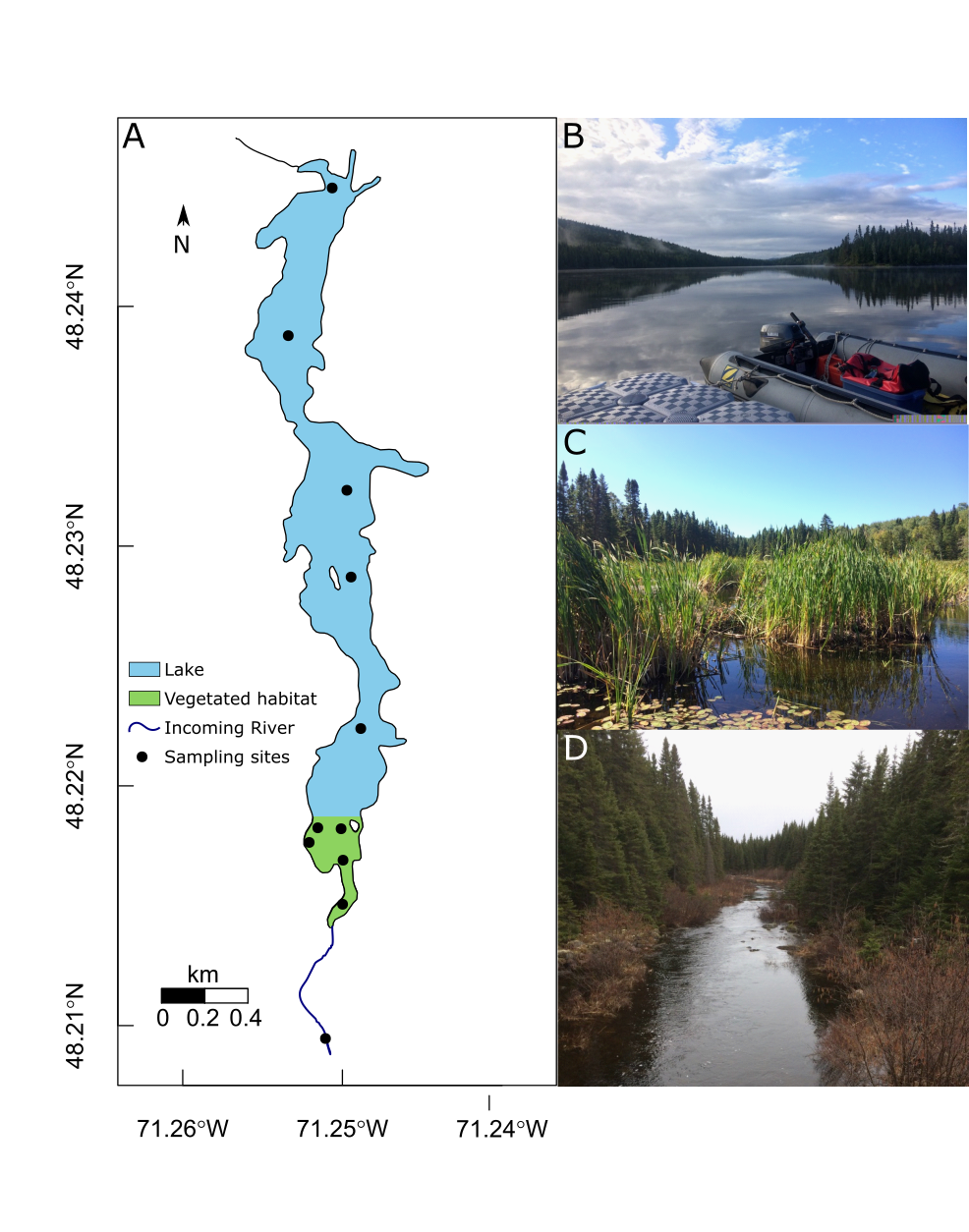


Fig. S3. Map of Lake Simoncouche detailing the different habitats and sampling sites (A). Map created in R with Statistics Canada open-access databases on Lakes and Rivers boundaries. Pictures of the lake (B), macrophyte vegetated habitat (C) and the incoming River (D). Photographs : Karelle Desrosiers and Alice Parkes.


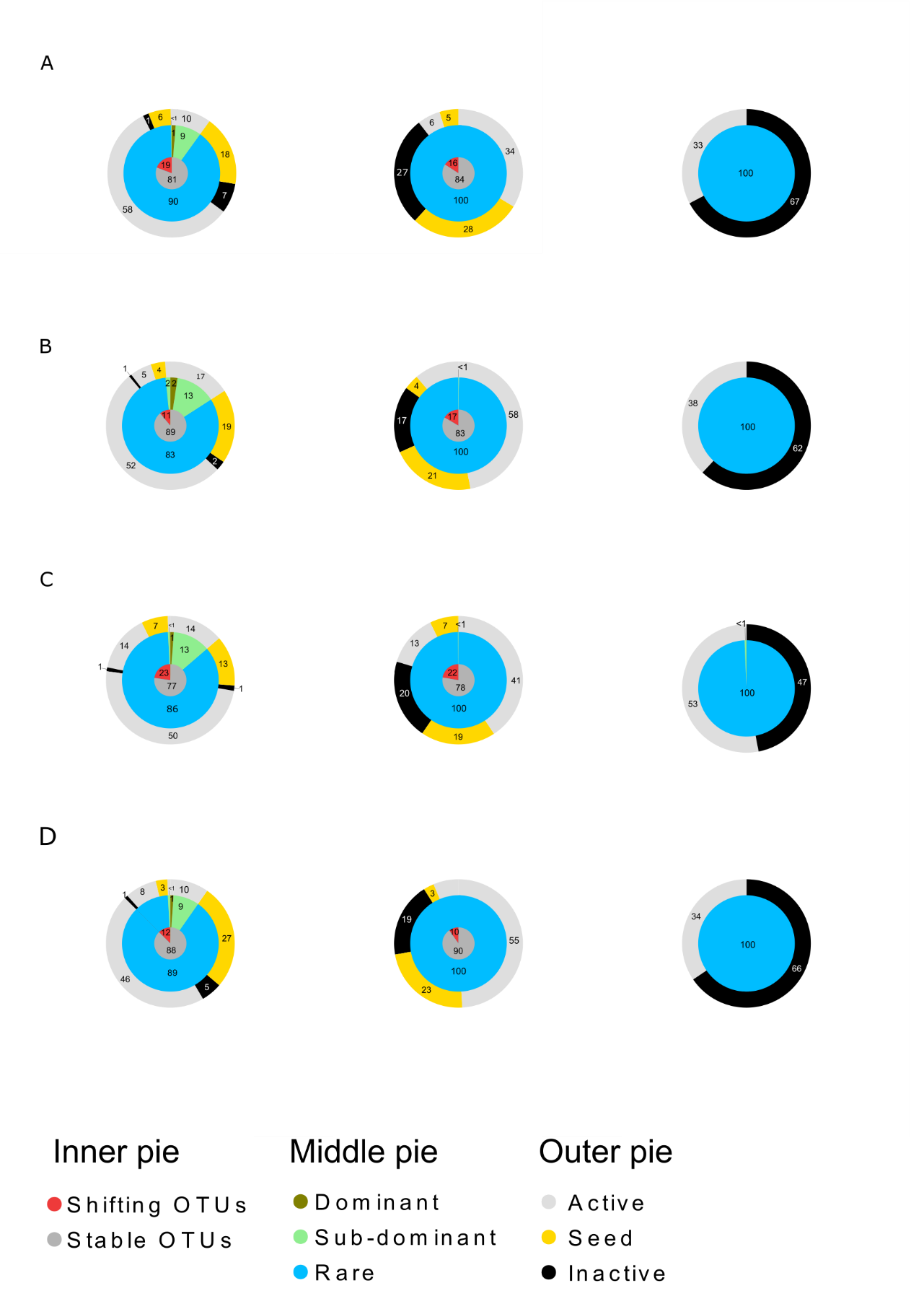


Fig. S4. Proportion of lake OTUs coming from the river (left pie), vegetated habitat (middle pie) and the lake (right pie) for the first (A), second (B), third (C) and fourth campaign (D). Only categories accounting for more than 0.1% of the OTUs are shown.


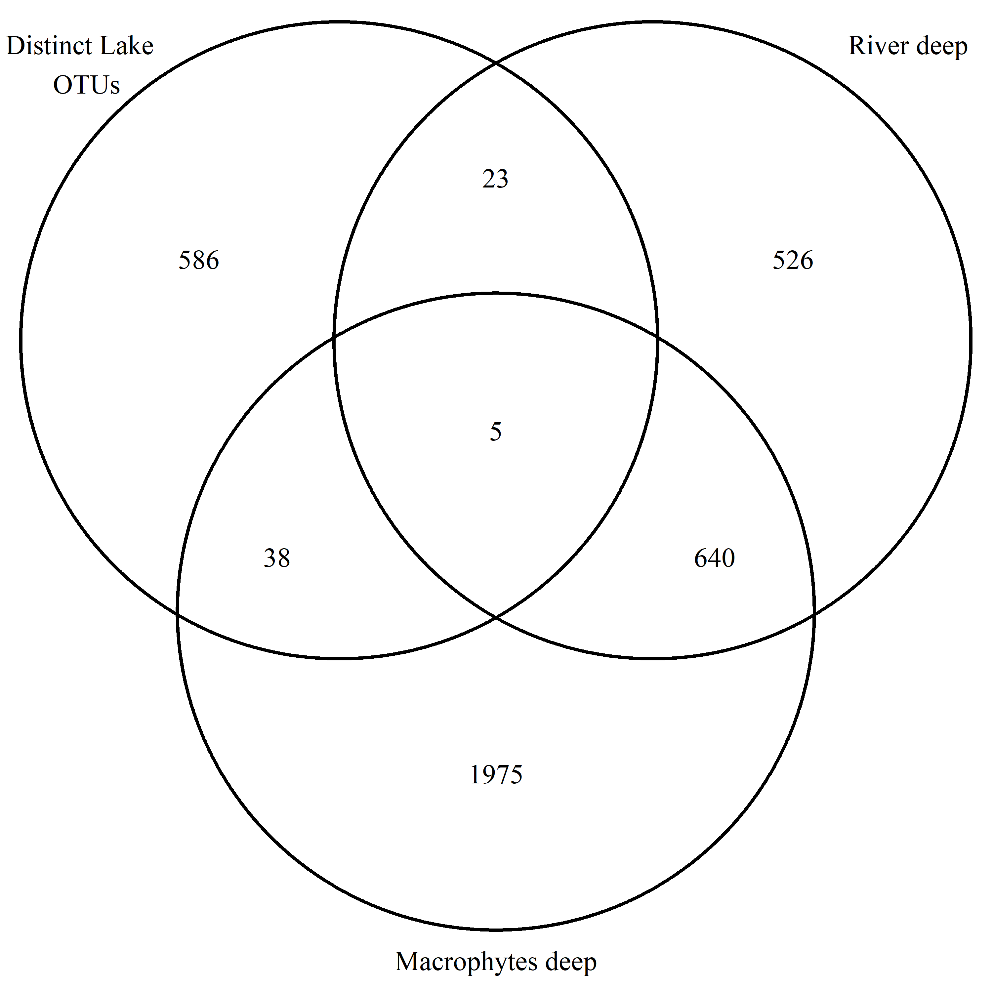


Fig. S5. Ven diagram showing the number of OTUs shared between the distinct OTUs that could only be found in the lake and the OTUs recovered from the deep sequencing of the River and Macrophyte samples during the second campaign.


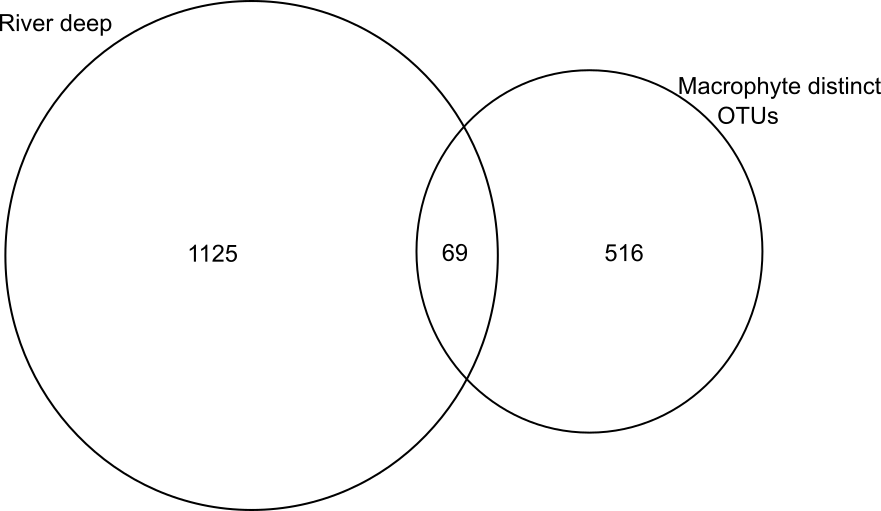


Fig. S6. Ven diagram showing the number of OTUs shared between the distinct OTUs that could only be found in the vegetated macrophyte-dominate habitat and the OTUs recovered from the deep sequencing of the River sample during the second campaign.
